# Epidemiological Study on *Mycoplasma pneumoniae* and *Chlamydia pneumoniae* Infection of Hospitalized Children in a Single Center During the COVID-19 Pandemic

**DOI:** 10.3389/fcimb.2022.843463

**Published:** 2022-03-21

**Authors:** Fengqing Cai, Xinyi Shou, Qing Ye

**Affiliations:** The Children’s Hospital, Zhejiang University School of Medicine, National Clinical Research Center for Child Health, National Children’s Regional Medical Center, Hangzhou, China

**Keywords:** COVID-19, *Mycoplasma pneumoniae*, *Chlamydia pneumoniae*, acute respiratory tract infection, epidemiological study

## Abstract

**Background:**

Since the outbreak of COVID-19, a series of preventive and control measures in China have been used to effectively curb the spread of COVID-19. This study aimed to analyze the epidemiological characteristics of *Mycoplasma pneumoniae* (*MP*) and *Chlamydia pneumoniae* (*CP*) in hospitalized children with acute respiratory tract infection during the COVID-19 pandemic.

**Methods:**

*MP* IgM antibody and *CP* IgM antibody were detected in all hospitalized children due to acute respiratory tract infection in the Children’s Hospital Affiliated to Zhejiang University from January 2019 to December 2020. These data were compared between 2019 and 2020 based on age and month.

**Results:**

The overall detection rate of *MP* and *CP* in 2020 was significantly lower than that in 2019 (*MP*: 21.5% vs 32.9%, *P*<0.001; *CP*: 0.3% vs 0.9%, *P*<0.001). This study found a 4-fold reduction in the number of children positive for *MP* and a 7.5-fold reduction in the number of children positive for *CP* from 2019 to 2020. The positive cases were concentrated in children aged >1 year old. In 2019, the positive rate of *MP* was detected more commonly in children 3 years of age or older than in younger children. In 2020, the higher positive rate of *MP* reached a peak in the 3- to 6-year age group (35.3%). *CP* was detected predominantly in children aged 6 years older in 2019 and 2020, with positive rates of 4.8% and 2.6%, respectively. Meanwhile, the positive rates of *MP* in 2019 were detected more commonly in July, August and September, with 47.2%, 46.7% and 46.3%, respectively. Nevertheless, the positive rates of *MP* from February to December 2020 apparently decreased compared to those in 2019. The positive rates of *CP* were evenly distributed throughout the year, with 0.5%-1.6% in 2019 and 0.0%-2.1% in 2020.

**Conclusions:**

A series of preventive and control measures for SARS-CoV-2 during the COVID-19 pandemic can not only contain the spread of SARS-CoV-2 but also sharply improve the infection of other atypical pathogens, including *MP* and *CP*.

## Introduction

COVID-19 pneumonia is characterized by acute respiratory infection, including fever, dry cough and fatigue. COVID-19 pneumonia is still in a pandemic state worldwide and is an urgent public health issue (Mallah et al., 2021). Since the outbreak of COVID-19, China has issued a series of preventive and control measures, including two overarching strategies, containment and suppression (Li et al., 2020; National Health Commission Of The People’s Republic Of, C., 2020). The core measures of containment are early finding infected people and actively treating in isolation, closely tracking and quarantining and reducing transmission, including staying at home, less aggregation and closing schools (Chen et al., 2021). Personal protection methods consist of wearing masks, washing hands frequently, keeping social distance, ventilation and gathering outside the house (Ju et al., 2021). These prevention and control measures refer to nonpharmaceutical interventions (Zhang et al., 2021a).

Zhejiang Province, located in East China, launched the first-level response to major public health emergencies on January 23, which was the first province in China to launch the “first-level emergency plan”. There were fewer children infected with COVID-19 in this area, including 36 children with COVID-19 in a previous multicenter study (Chen et al., 2020), and only one infant with COVID-19 was treated in our hospital. Recently, a few studies found that nonpharmaceutical interventions aimed at COVID-19 not only contain the spread of COVID-19 but also markedly reduce influenza virus infection (Fricke et al., 2021) and the transmission of other respiratory viruses, such as respiratory syncytial virus and adenovirus (Ye and Liu, 2021).

Acute respiratory tract infection is a common infectious disease in children. *Mycoplasma pneumoniae* (*MP*) and *Chlamydia pneumoniae* (*CP*) are atypical pathogens of acute respiratory tract infections in children. In particular, *MP* pneumonia accounts for 32.4%-39.5% of children’s community-acquired pneumonia (Ning et al., 2017; Gao LW et al., 2019). It is common in outpatients and hospitalized children and is mainly transmitted through the respiratory tract. This study aimed to analyze the epidemiological characteristics of *MP* and *CP* in hospitalized children with acute respiratory tract infection, which is transmitted by respiratory droplets similar to SARS-CoV-2, during the COVID-19 pandemic (Lansbury et al., 2020; Oliva et al., 2020; Zhu et al., 2020).

## Methods

### Study Subjects

The retrospective study included all children hospitalized due to acute respiratory tract infection in the Children’s Hospital Affiliated to Zhejiang University from January 2019 to December 2020. Demographic data, such as age, gender, and the patient’s clinical manifestations, were obtained from the electronic medical records. All enrolled children conformed to the following criteria: (1) one or more respiratory symptoms (cough, sore throat, combined with a body temperature > 37.5°C) (McCracken, 2001) and (2) children aged younger than 18 years. The exclusion criteria of this study were as follows: (1) children infected with COVID-19; (2) children with malignant tumors or congenital pulmonary airway obstruction; and (3) children with a recurrent chronic respiratory infection. All the children were divided into five age groups: under 28 days (0–28 d), 1-12 months (1-12 m), 1-3 years (1-3 y), 3-6 years (3-6 y) and six years older (> 6 y). The detection rate of pathogens was also compared by month.

### Detection of Pathogens

After admission, blood was collected with a heparin anticoagulant tube and then centrifuged for 5 minutes at 2500 r/min. Centrifuged serum was used for detection. *MP* IgM and *CP* IgM antibodies were detected by a two-step indirect method of direct chemiluminescence technology (iFlash3000, YHLO, Shenzhen, China) using a commercial kit (YHLO, Biotechnology Co., Ltd., China). The steps of detection were as follows. The first step of incubation was that *MP* or *CP* IgM in the sample reacted with the corresponding antigen coated on superparamagnetic particles to form antigen-antibody complexes. Magnetic particles were adsorbed to the reaction tube wall under the action of a magnetic field, and unbound substances were washed away by the cleaning solution. The second incubation step was that mouse anti-human IgM labeled with acridine was added to the reaction tube to form an antigen-antibody-double antibody complex. Unbound substances were washed away again. Preexcitation solution and excitation solution were added to the reaction mixture, and then the relative luminous intensity (RLU) of the mixture was detected by the optical system of the tester. The amount of pathogen IgM in the sample was proportional to the RLU. The cutoff index (COI) value was automatically calculated according to the RLU value of each sample by the tester. The relative luminous intensity was compared with the cutoff value calculated by the corresponding IgM calibrator. When the COI < 0.9, the IgM antibody was negative. When the COI was between 0.9-1.1, the result needed to be rechecked or comprehensively judged. When the COI ≥ 1.1, the IgM antibody was positive.

The final diagnosis of *M. pneumoniae* and *C. pneumoniae* infection in our manuscript combined serological IgM antibody with the patient’s clinical symptoms, other laboratory indicators (leukocytes, hypersensitive C-reactive protein, cytokines, etc.) and imaging data (Meyer Sauteur et al., 2016).

### Statistical Analysis

All the data were analyzed using SPSS version 26.0 software (IBM Corp., Armonk, N.Y., USA). Categorical variables were analyzed using the chi-squared test or Fisher’s exact test. A *P* value <0.05 was considered statistically significant.

## Results

### Patient Characteristics

From January 2019 to December 2020, a total of 18833 children were admitted to our hospital due to acute respiratory infection, including 13621 cases in 2019 and 5212 cases in 2020. The total number of patients in 2020 was significantly lower than that in 2019 (*P*<0.001). Among all the enrolled children, 10922 were male and 7911 were female, with a male to female ratio of 1.38:1. There was no significant difference in sex between patients in 2019 and those in 2020. In the five age groups, most patients were aged 1-3 years (7023, 37.3%), followed by children aged 1-12 months (5510, 29.3%). (**Table 1**)

**Table 1:** Patient characteristics and detection of *Mycoplasma pneumoniae* and *Chlamydia pneumoniae* between 2020 (during the COVID-19 pandemic) and 2019 (before the COVID-19 pandemic).

	2019 (n=13621)	2020 (n=5212)	*χ^2^* value	*P* value
**Characteristics, n (%)**				
Age				
0-28 d	729 (5.4)	345 (6.7)	11.26	0.001
1-12 mo	3795 (27.9)	1715 (32.9)	46.33	<0.001
1-3 y	5055 (37.1)	1968(37.8)	91.31	<0.001
3-6 y	2378 (17.5)	609 (11.7)	94.17	<0.001
>6 y	1664 (12.2)	573 (11.0)	5.38	0.020
Gender				
Male	7856 (57.7)	3066 (58.8)	2.047	0.153
Female	5765 (42.3)	2146 (41.2)		
**Detection of the pathogen, n (%)**				
* MP* positive specimens	4476 (32.9)	1118 (21.5)	235.05	<0.001
* CP* positive specimens	128 (0.9)	17 (0.3)	18.57	<0.001
**Total**	4604 (33.8)	1135 (21.8)	257.23	<0.001

### Overall Detection of *MP* and *CP*

In 2019 (before the COVID-19 pandemic), *MP* was detected in 32.9% of patients (4476/13621), and *CP* was detected in 0.9% (128/13621). However, in 2020 (during the COVID-19 pandemic), *MP* was detected in 21.5% of patients (1118/5212), and *CP* was detected in 0.3% (17/5212). We found a 4-fold reduction in the number of children positive for *MP* and a 7.5-fold reduction in the number of children positive for *CP* from 2019 to 2020. The positive cases were concentrated in children aged >1 year old. The overall detection rate of *MP* and *CP* in 2020 was significantly lower than that in 2019 (χ^2^ = 235.05, *P*<0.001; χ^2^ = 18.57, *P*<0.001). (**Table 1**).

### Age Distribution

**Figure 1:**
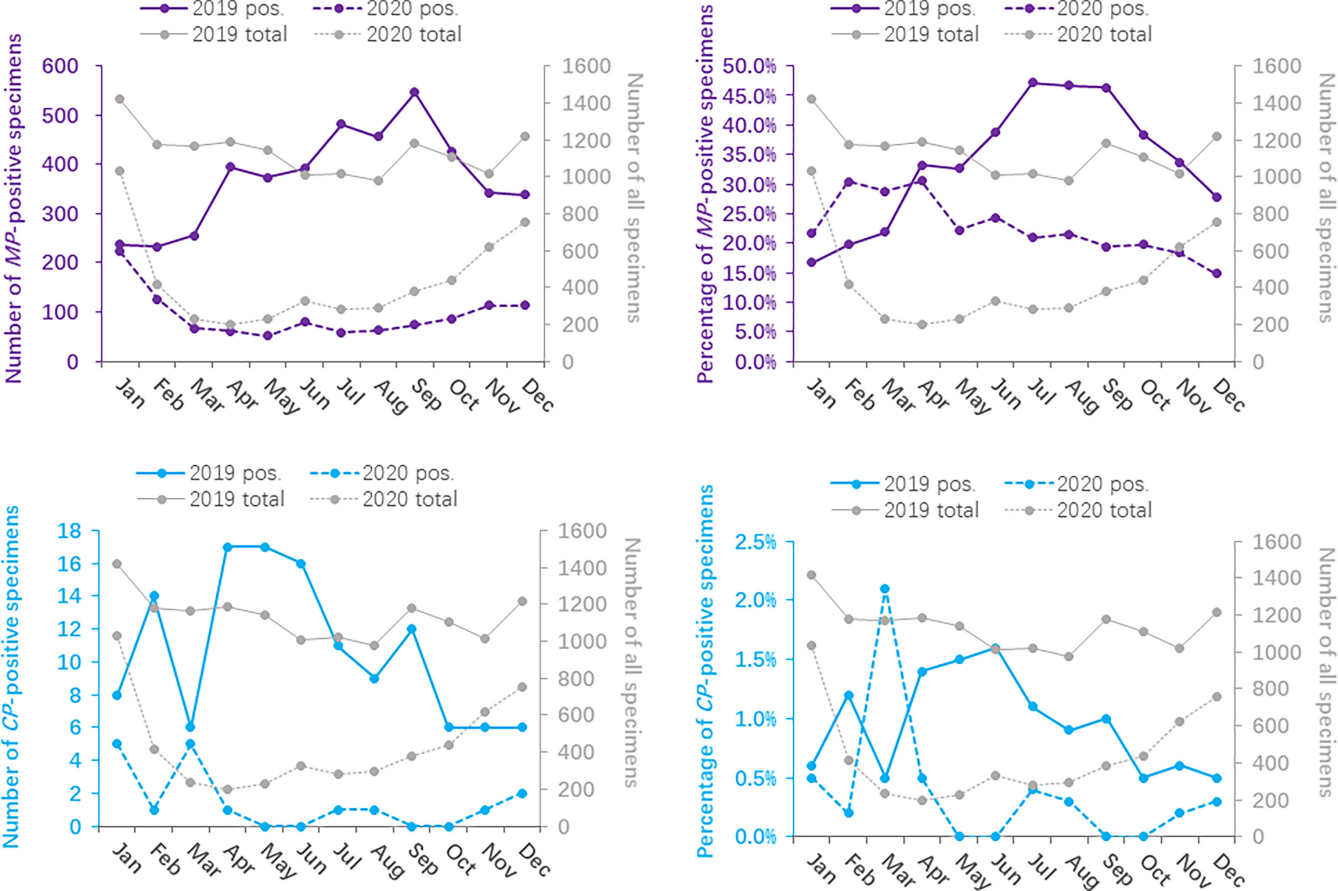
Distribution of *Mycoplasma pneumoniae-* and *Chlamydia pneumoniae*-positive specimens based on age between 2020 (during the COVID-19 pandemic) and 2019 (before the COVID-19 pandemic).

The age group distributions of the positivity detection rates of *MP* and *CP* based on 2019 and 2020 are shown in **Figure 1**. The number of *MP*-positive patients reached a peak in the 1-3 year age group, with peaks of 2068 in 2019 and 548 in 2020. However, in 2019, the positive rate of *MP* was detected more commonly in children 3 years of age or older (47.8% in the age of 3-6 years, 50.7% in the age of >6 years) than in younger children (40.9% in the age of 1-3 years, 11.3% in the age of 1-12 months, 0.1% in the age of 0-28 days). In 2020, the higher positive rate of *MP* reached a peak in the 3- to 6-year age group (35.3%). *MP*-positive numbers in children aged 1-12 months decreased more than two times from 2019 to 2020 (427 vs. 190), and *MP*-positive numbers in children aged 3 years decreased more than 5 times from 2019 to 2020 (1136 vs. 215 in children aged 3-6 years; 844 vs. 164 in children aged 6 years). Nevertheless, the positive rate of *MP* in children older than 1 year was significantly decreased in 2020 (*P*<0.001). *CP* was detected predominantly in children aged 6 years older in 2019 and 2020, with positive numbers of 80 and 14, respectively. In children aged 6 years old, the positive rate of *CP* in 2020 was lower than that in 2019 (χ^2^ = 5.919, *P*=0.015). (**Table 2**)

**Table 2:** The overall positive rate of *Mycoplasma pneumoniae* and *Chlamydia pneumoniae* based on age between 2020 (during the COVID-19 pandemic) and 2019 (before the COVID-19 pandemic).

Age	2019	2020	*χ^2^* value	*P*-value
**0-28d**				
*** MP***	1/729 (0.1%)	1/345 (0.3%)	0.289	0.591
*** CP***	0/729 (0.0%)	0/345 (0.0%)	/	/
** Total**	1 /729(0.1%)	1/345 (0.3)	0.289	0.591
**1-12mo**				
*** MP***	427/3795 (11.3%)	190/1715 (11.1%)	0.036	0.851
*** CP***	1/3795 (0.1%)	2/1715 (0.1%)	1.769	0.184
** Total**	428/3795 (11.4%)	192/1715 (11.2%)	0.008	0.928
**1-3y**				
*** MP***	2068/5055 (40.9%)	548/1968 (27.8%)	103.436	<0.001
*** CP***	16/5055 (0.3%)	0/1968 (0.0%)	6.243	0.012
** Total**	2084/5055 (41.2%)	548/1968 (27.8%)	108.242	<0.001
**3-6y**				
*** MP***	1136/2378 (47.8%)	215/609 (35.3%)	30.422	<0.001
*** CP***	31/2378 (1.3%)	1/609 (0.2%)	5.939	0.015
** Total**	1167/2378 (49.1%)	216/609 (35.5%)	36.104	<0.001
**>6y**				
*** MP***	844/1664 (50.7%)	164/573 (28.6%)	102.806	<0.001
*** CP***	80/1664 (4.8%)	14/573 (2.6%)	5.919	0.015
** Total**	924/1664 (55.5%)	178/573 (31.2%)	102.061	<0.001

Data were expressed as the positive number/the total number (%).

During the COVID-19 epidemic, the number of *MP* infections in children except for infants aged 0-28 days decreased significantly compared to that in 2019. The *MP*-positive rate of children aged 3 years old reached a peak in 2019, while only children aged 3-6 years old showed a small peak in 2020. *CP* infections mainly occurred in children aged 6 years older.

### Season Distribution

**Figure 2:**
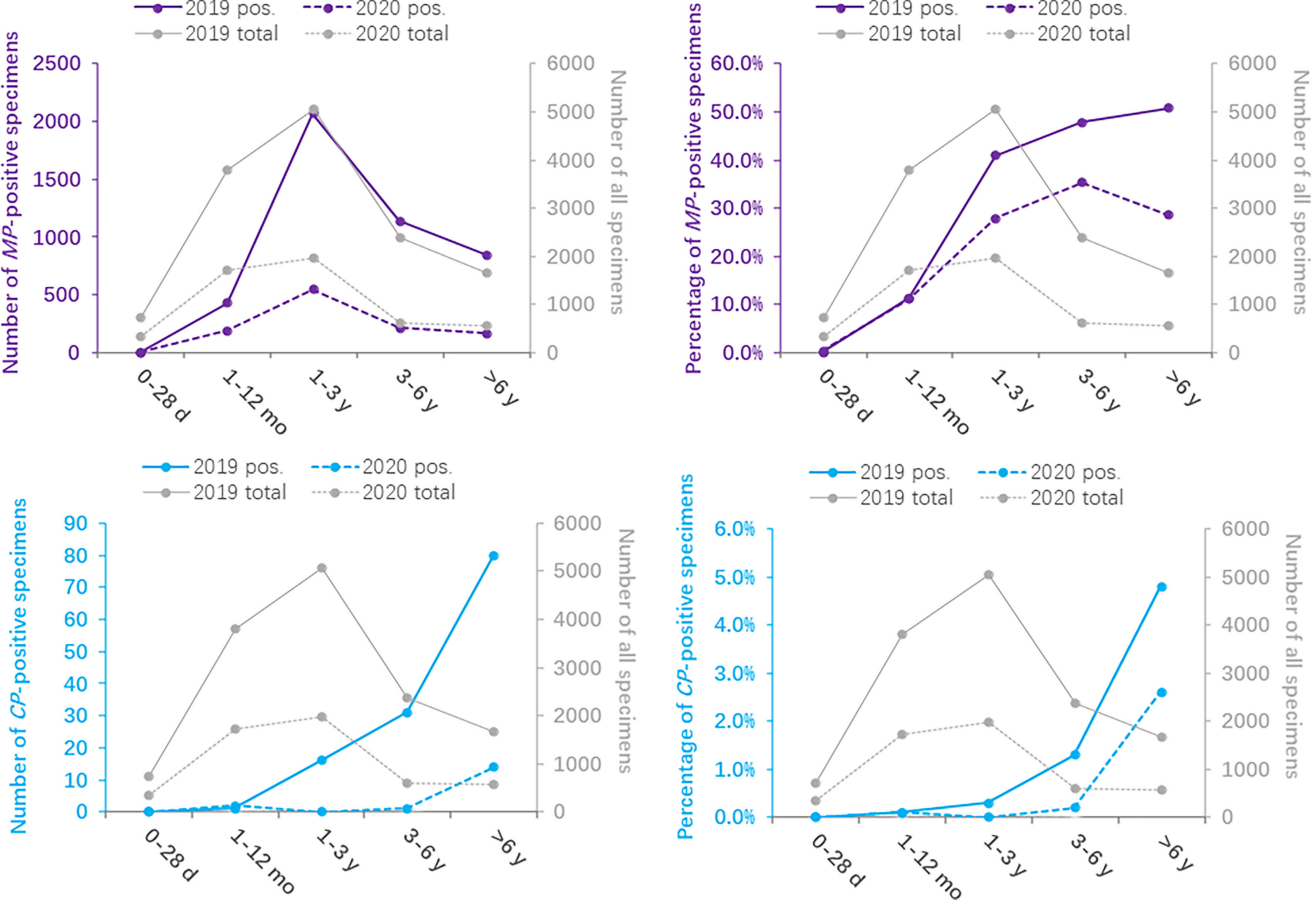
Distribution of *Mycoplasma pneumoniae-* and *Chlamydia pneumoniae-*positive specimens based on months between 2020 (during the COVID-19 pandemic) and 2019 (before the COVID-19 pandemic).

The monthly distribution of the positivity detection rate of *MP* and *CP* based on 2019 and 2020 is shown in **Figure 2**. The number of *MP*-positive patients in 2019 reached a peak in September (546/1178), followed by July (481/1020) and August (457/978), whereas the peak number of *MP*-positive patients in 2020 was in January (223/1032). Meanwhile, the positive rates of *MP* in 2019 were detected more commonly in July, August and September, with 47.2%, 46.7% and 46.3%, respectively. Nevertheless, the positive rates of *MP* from February to December 2020 apparently decreased compared to those in 2019. The numbers of *MP*-positive cases were not significantly different between January 2019 and January 2020 (237 vs 223, *P*>0.05). The positive rates of *CP* were evenly distributed throughout the year, with 0.5%-1.6% in 2019 and 0.0%-2.1% in 2020. (**Table 3**)

**Table 3:** Detection of *Mycoplasma pneumoniae* and *Chlamydia pneumoniae* based on months between 2020 (during the COVID-19 pandemic) and 2019 (before the COVID-19 pandemic).

Month	MP		*χ^2^* value	*p*-value	CP		*χ^2^* value	*p*-value
	2019	2020			2019	2020		
**January**	237/1419 (16.7%)	223/1032 (21.6%)	12.45	<0.001	8/1419 (0.6%)	5/1032 (0.5%)	0.07	0.799
**February**	233/1177 (19.8%)	126/415 (30.4%)	19.61	<0.001	14/1177 (1.2%)	1/415 (0.2%)	2.96	0.085
**March**	256/1167 (21.9%)	67/233 (28.8%)	5.10	0.024	6/1167 (0.5%)	5/233 (2.1%)	0.02	0.879
**April**	394/1186 (33.2%)	61/200 (30.5%)	0.58	0.448	17/1186 (1.4%)	1/200 (0.5%)	1.16	0.281
**May**	373/1141 (32.7%)	51/230 (22.2%)	9.91	0.002	17/1141 (1.5%)	0/230 (0.0%)	3.47	0.062
**June**	392/1011 (38.8%)	80/329 (24.3%)	22.74	<0.001	16/1011 (1.6%)	0/329 (0.0%)	5.27	0.022
**July**	481/1020 (47.2%)	59/282 (20.9%)	62.64	<0.001	11/1020 (1.1%)	1/282 (0.4%)	1.27	0.260
**August**	457/978 (46.7%)	63/293 (21.5%)	59.35	<0.001	9/978 (0.9%)	1/293 (0.3%)	0.97	0.325
**September**	546/1178 (46.3%)	74/381 (19.4%)	87.15	<0.001	12/1178 (1.0%)	0/381 (0.0%)	3.91	0.048
**October**	426/1109 (38.4%)	87/439 (19.8%)	49.01	<0.001	6/1109 (0.5%)	0/439 (0.0%)	2.38	0.123
**November**	343/1017 (33.7%)	114/621 (18.4%)	45.28	<0.001	6/1017 (0.6%)	1/621 (0.2%)	1.67	0.197
**December**	338/1218 (27.8%)	113/757 (14.9%)	43.56	<0.001	6/1218 (0.5%)	2/757 (0.3%)	0.60	0.437

Data were expressed as the positive number/the total number (%).

Before the COVID-19 pandemic, *MP*-positive infections were detected more commonly in July, August and September, whereas no such regular seasonal variation was found in 2020. The study based on month showed a low prevalence of *CP*-positive infections in 2019 and 2020.

## Discussion

Since COVID-19, caused by severe acute respiratory syndrome coronavirus 2 (SARS-CoV-2), was first discovered in mid-December 2019 in Wuhan, China, a series of nonpharmaceutical interventions have been promptly taken to control the transmission of SARS-CoV-2 in China (Zhang et al., 2021a). Apart from controlling the spread of COVID-19, the epidemiology of other atypical pathogens, such as *MP* and *CP*, has undergone tremendous changes at the same time due to these nonpharmaceutical interventions. In our study, the number of children hospitalized due to acute respiratory tract infection in 2020 declined by 59.5% compared to that in 2019. Compared to 2019, the total positive rate of atypical pathogens decreased in 2020, with an emphasis on the positive rate of *MP* detection from 32.9% to 21.5%. This was consistent with previous results obtained in other regions of China (Li et al., 2021), in which the number of positive *MP* cases significantly decreased in 2020 for the public health response to COVID-19 (Zhang et al., 2021b). The positive rate of *CP* among children with acute respiratory infection decreased from 0.9% in 2019 to 0.3% in 2020, which was significantly lower than that of *MP* infection. Similar results were shown in a 5-year multicenter epidemiological study (Luo et al., 2021). In our present study, the positive rate of *MP* detection in children was significantly higher than that in adults (19%) (Chen et al., 2019), probably due to the mature immune system, paying more attention to hygiene and maintaining social distance at work. The decrease in the overall positive rate of atypical pathogens showed the significant additional effect of these nonpharmaceutical interventions in response to the COVID-19 epidemic.

In our study, children aged 1-3 years were most likely to develop acute respiratory tract infection symptoms in both 2019 and 2020. In addition, the positive rate of *MP* was detected more commonly in children 3 years of age or older than in younger children, which was corroborated by previous epidemiological studies (Jain et al., 2015; Schildgen et al., 2018; Liu et al., 2020). However, the positive rate of *MP* in our study is different from that in other previous studies (Gao LW et al., 2019; Jiang et al., 2020), which may be due to differences in *MP* detection methods and enrolled populations. Chemiluminescence was used to detect IgM antibodies of serum *MP* in our study, whereas atypical pathogen DNA was detected in nasopharyngeal samples by a polymerase chain reaction in some studies (Del Valle-Mendoza et al., 2017; Jiang et al., 2020; Kumar et al., 2020), or *MP* antibody was detected by the passive agglutination method (Gao CH et al., 2019; Gao LW et al., 2019). PCR-based techniques are now considered more reliable, however, this method also has limitations, such as low sensitivity and positive predictive values (Nilsson et al., 2008; Chang et al. 2014; Kurkela et al. 2019). So, each method, whether serological test or PCR method, has its advantages and disadvantages. Diagnosis needs to combined laboratory examination with clinical manifestation (Meyer Sauteur et al. 2016).

Among the five age groups, the positive detection rate of *MP* in children over 3 years old in 2019 was higher than that in other age groups, while the infection rate of infants younger than 28 days was very low. This was due to cross-infection among kindergarten and school-age children, carefully taking care by parents and hardly contacting outsiders for infants within 28 days. Interestingly, the positive detection rate of *MP* in children over 3 years old improved significantly in 2020, which is inseparable from the strict COVID-19 prevention and control policy. The policies included delaying school opening in spring and wearing masks, hand hygiene and social distancing.

We found that the number of *MP*-positive patients in 2019 reached a peak in September, followed by July and August, whereas the peak number of *MP*-positive patients in 2020 was in January. Previous studies have reported that the climate environment can significantly affect the transmission of *MP* pathogens (Wright et al., 1969; Tian et al., 2017). Our previous study suggested that *MP* infection significantly correlated with temperature (Tian et al., 2017). The optimum growth temperature of *MP* is 37°C. In China, July, August and September are the hottest months every year, and *MP* grows best at these higher temperatures. Since Zhejiang initiated the 1-level emergency response on January 23, 2020, the number of children hospitalized due to acute respiratory tract infection decreased sharply in February 2020, but the positive rate was still 30.4%. This may be because the number of *MP*-positive patients did not decrease as fast as the total number of hospitalizations. The numbers of *MP*-positive patients and the total number of hospitalizations from March to December 2020 were obviously lower than those in 2019, which was consistent with previous results found in Chengdu, China (Zhang et al., 2021b). Although the positive rate of *CP* was low in both 2019 and 2020, the number of *CP*-positive patients monthly in 2020 was still significantly decreased compared with that in 2019. A low prevalence of *CP* infections was also found in other countries, such as Japan (Oishi et al., 2020; Ishimaru et al., 2021), or other regions in China (Li et al., 2021; Luo et al., 2021). Due to the strict prevention and control policy for COVID-19 in China, the positive rate of *CP* reached a lower degree (0.0%-0.5%) from April to December 2020.

Many studies have reported that atypical pathogens can cause respiratory diseases and may induce secondary diseases such as skin lesions, especially urticaria (De Luigi et al., 2021), Fuchs’ syndrome, varicella-like eruptions, Henoch-Schonlein syndrome (Terraneo et al., 2015), acute kidney injury and myositis (Simoni et al., 2020). Therefore, an enormous reduction in the number of children positive for *MP* and *CP* had great significance in reducing the burden of respiratory diseases and may reduce the occurrence of these secondary diseases.

Overall, these strict prevention and control measures to effectively curb the spread of atypical pathogens may be due to multiple factors. First, wearing masks and social distancing effectively blocked the transmission of atypical pathogens from infected people. During the COVID-19 epidemic, a study proved that masks could prevent infectious aerosols produced by infected people (Ju et al., 2021). Similarly, social distancing can effectively reduce aerosol transmission between close contacts (Kucharski et al., 2020). Second, staying at home, less aggregation, and closing schools can control the spread of atypical pathogens between children at the source. Third, frequent hand hygiene may eliminate atypical pathogens to a certain extent, which was also sensitive to 75% alcohol.

Nevertheless, our study was subject to some limitations. First, this is a single-center study, even though this hospital is the largest children’s hospital in Zhejiang Province, China. The prevalence of atypical pathogens may differ from other provinces in China with different climates, economic levels and lifestyles. Second, the pathogens detected in our study included only *MP* and *CP* and did not analyze other respiratory viruses and bacterial pathogens. Because this was a retrospective study, detailed data on other pathogens were lacking.

## Conclusion

In conclusion, this is the first study to analyze the impact of preventive and control measures for SARS-CoV-2 on the transmission of *MP* and *CP* infection during the COVID-19 pandemic in Zhejiang Province, China. A series of preventive and control measures for SARS-CoV-2 during the COVID-19 pandemic not only contain the spread of SARS-CoV-2 but also sharply improve the infection of other atypical pathogens, including *MP* and *CP*.

## Data Availability Statement

The original contributions presented in the study are included in the article/supplementary material. Further inquiries can be directed to the corresponding author.

## Ethics Statement

The study was approved by the medical ethics committee of the Children’s Hospital of Zhejiang University School of Medicine (Hangzhou, China). Written informed consent to participate in this study was provided by the participants’ legal guardian/next of kin.

## Author Contributions

QY conceived and designed the study and took responsibility for the integrity of the data and the accuracy of the data analysis. FC contributed to the writing of the report. All authors contributed to data acquisition, analysis, or interpretation and reviewed and approved the final version.

## Funding

This study was supported by the Natural Science Foundation of Zhejiang Province (LY22H050001), the Key Project of Provincial Ministry Construction, Health Science and Technology Project Plan of Zhejiang Province (WKJ-ZJ-2128), Key Laboratory of Women’s Reproductive Health Research of Zhejiang Province (No. ZDFY2020-RH-0006), the National Natural Science Foundation of China (Grant/Award Number: U20A20351),and Key Research and Development Plan of Zhejiang Province (Grant/Award Number: 2021C03079).

## Conflict of Interest

The authors declare that the research was conducted in the absence of any commercial or financial relationships that could be construed as a potential conflict of interest.

## Publisher’s Note

All claims expressed in this article are solely those of the authors and do not necessarily represent those of their affiliated organizations, or those of the publisher, the editors and the reviewers. Any product that may be evaluated in this article, or claim that may be made by its manufacturer, is not guaranteed or endorsed by the publisher.
